# Supplementary material for: Assessment of dried blood spots for DNA methylation profiling
Source: Wellcome Open Res. 2019 Mar 6;4:44. [Version 1] doi: 10.12688/wellcomeopenres.15136.1 (PMC6446498; doi:10.12688/wellcomeopenres.15136.1)
Supplement: Peer review history

# Peer review: Reviewer response for version 1

**DOI:** 10.21956/wellcomeopenres.16515.r35009

**Published:** 2019-04-01

**Authors:** JiHoon Eric Joo

**Affiliations:** 1 Department of Clinical Pathology, The University of Melbourne, Parkville, Australia; 2 University of Melbourne Centre for Cancer Research, The University of Melbourne, Parkville, Australia

This manuscript describes that the utility of DNA extracted from archival DBS on Whatman FTA for measuring genome-wide DNA methylation. The authors have conducted an in-depth analysis to assess the reliability and feasibility of using such DNA materials for genome-wide DNA methylation analysis and showed a good correlation with same blood samples stored in -20ºC over long periods of time. The manuscript is very well presented and free from errors. The discussion section is well-written and appropriately cited and compared with existing literature's with similar findings. I have a small number of minor suggestions that could improve the manuscript further.

1. All three figures (Figure 1-3) are in poor resolution.
2. It seems like only current smoking status has been used in the analysis? How about former smokers and its effect on DNA methylation at the *AHRR *or other loci?
3. Although it was only a slight variation in signals in methylated/unmethylated probes between DBS and EDTA blood DNA, could this introduce false positives/negatives? Is there anything obvious about these sites (e.g. low-density CpG, promoter)? Though it’s subtle, it seems too systemic to be ignored. This could be related to conversion efficiency? Could the authors investigate this further by looking at other additional probes than control probes?
4. How was the age analysis performed? Was age transformed into splines or treated as a numeric variable?
5. In addition to looking at age-associated probes, the authors also try calculating DNAmAge using Horvath’s or Hannam’s epigenetic clock algorithms and describes which DNA samples show higher correlation with the chronological age, if they show any differences.
6. Please provide some more description on how the relationship between DNA methylation and storage time was calculated? Was storage time was correlated with years or days? Was there any difference in DNA quality (i.e. molecular weight)?
7. Why different DNA extraction methods (kits) were used for DBS and WB?
8. Please specify explicitly how this investigation is distinct from other similar studies mentioned (i.e. Joo et al., 2013 ^1^ and Hollegard et al.,2013 ^2^) in discussion. Is this about using the FTA card or applying the results to identify smoking and ageing-associated CpG probes?

I have read this submission. I believe that I have an appropriate level of expertise to confirm that it is of an acceptable scientific standard.

## References

[1] JooJEWongEMBagliettoLJungCHTsimiklisHParkDJWongNCEnglishDRHopperJLSeveriGGilesGGSoutheyMC: The use of DNA from archival dried blood spots with the Infinium HumanMethylation450 array. *BMC Biotechnol*.2013;13: 10.1186/1472-6750-13-23 23 10.1186/1472-6750-13-23 23497093PMC3610215

[2] HollegaardMVGrauholmJNørgaard-PedersenBHougaardDM: DNA methylome profiling using neonatal dried blood spot samples: a proof-of-principle study. *Mol Genet Metab*.2013;108(4) : 10.1016/j.ymgme.2013.01.016 225-31 10.1016/j.ymgme.2013.01.016 23422032

# Peer review: Reviewer response for version 1

**DOI:** 10.21956/wellcomeopenres.16515.r35011

**Published:** 2019-03-27

**Authors:** Jeffrey M. Craig

**Affiliations:** 1 Centre for Molecular and Medical Research (C-MMR), School of Medicine, Deakin University, Geelong, Vic, Australia

The manuscript presents evidence to support the usage of aged dried blood spots as an adequate substitute for fresh or frozen EDTA blood samples. As dried blood spots are easier to collect and cheaper to store than fresh blood, and are stored routinely at birth in most countries, the knowledge generated is quite useful and adds a little novelty to the literature. The manuscript is generally well written, easy to follow and scientifically sound. However, it is not as novel as it claims. 

 Firstly, the authors state that their study is the first methylome-wide study to compare fresh and archived dried blood spots. This is not the case; the following studies have indeed done something the same or very similar:

 Aberg KA et al, High quality methylome-wide investigations through next-generation sequencing of DNA from a single archived dry blood spot ^1^.

 Dugué PA et a,l Reliability of DNA methylation measures from dried blood spots and mononuclear cells using the HumanMethylation450k BeadArray ^2^.

 Forest M et al, Agreement in DNA methylation levels from the Illumina 450K array across batches, tissues, and time ^3^. 

 Secondly, the authors do not cite one previous methylome –wide study of smoking exposur in DNA from dried blood spots; can they please do this:

 Li S et al, Causal effect of smoking on DNA methylation in peripheral blood: a twin and family study ^4^.

 In addition, the authors have also not cited the earliest recorded methylome-wide study in DNA from aged DBS. Can they please do this?

 Cruickshank MN et al, Analysis of epigenetic changes in survivors of preterm birth reveals the effect of gestational age and evidence for a long term legacy ^5^.

I have read this submission. I believe that I have an appropriate level of expertise to confirm that it is of an acceptable scientific standard.

## References

[1] AbergKAXieLYNerellaSCopelandWECostelloEJvan den OordEJ: High quality methylome-wide investigations through next-generation sequencing of DNA from a single archived dry blood spot. *Epigenetics*.2013;8(5) : 10.4161/epi.24508 542-7 10.4161/epi.24508 23644822PMC3741224

[2] DuguéPEnglishDMacInnisRJungCBassettJFitzGeraldLWongEJooJHopperJSoutheyMGilesGMilneR: Reliability of DNA methylation measures from dried blood spots and mononuclear cells using the HumanMethylation450k BeadArray. *Scientific Reports*.2016;6(1) : 10.1038/srep30317 10.1038/srep30317 27457678PMC4960587

[3] ForestMO'DonnellKJVoisinGGaudreauHMacIsaacJLMcEwenLMSilveiraPPSteinerMKoborMSMeaneyMJGreenwoodCMT: Agreement in DNA methylation levels from the Illumina 450K array across batches, tissues, and time. *Epigenetics*.2018;13(1) : 10.1080/15592294.2017.1411443 19-32 10.1080/15592294.2017.1411443 29381404PMC5837078

[4] LiSWongEMBuiMNguyenTLJooJEStoneJDiteGSGilesGGSafferyRSoutheyMCHopperJL: Causal effect of smoking on DNA methylation in peripheral blood: a twin and family study. *Clin Epigenetics*.2018;10: 10.1186/s13148-018-0452-9 18 10.1186/s13148-018-0452-9 29456763PMC5810186

[5] CruickshankMNOshlackAThedaCDavisPGMartinoDSheehanPDaiYSafferyRDoyleLWCraigJM: Analysis of epigenetic changes in survivors of preterm birth reveals the effect of gestational age and evidence for a long term legacy. *Genome Med*.2013;5(10) : 10.1186/gm500 96 10.1186/gm500 24134860PMC3978871
